# Supplementary material for: Biostimulants as an Alternative to Improve the Wine Quality from Vitis vinifera (cv. Tempranillo) in La Rioja
Source: Plants (Basel). 2022 Jun 16;11(12):1594. doi: 10.3390/plants11121594 (PMC9229063; doi:10.3390/plants11121594)
Supplement: Supplementary file 1 [file plants-11-01594-s001.zip › plants-1728186-supplementary.pdf]

Table S1. Additional vineyard treatments and damages during the seasons 2018 and 2019.

Date	*Plague*	*Commercial Product*	*Dose*	*Observations*
**1-3-18**	Grass	PATTEN GREEN	1 L/ha	
**8-5-18**	Oidium	AIRUS	170 cc/ha	
**9-5-18**	Dust Mites	ENVIDOR	100 cc/ha	
**10-5-18**	Mildew	ESTUDER	1.5 kg/ha	
**20-6-18**	Mildew	POPPIER PRO	2 kg/ha	
**21-6-18**	Oidium	LUNA EXPERIENCE	400 cc/ha	Poor setting in Greetnal, SoilExpert, and Basofoliar in Subplot A and Basofoliar in Subplot B
**2-7-18**	Mildew	POPPIER PRO	2 kg/ha	
**3-7-18**	Mildew	ASBELTO	2 kg/ha	
**3-7-18**	Oidium	COLLIS	400 cc/ha	
**17-7-18**	Mildew	ELECTIS	1 L/ha	Two strong storms of 26L/h and 20L/h that affected Greetnal and Basofoliar in Subplot B and Control, Greetnal, Basofoliar, and SoilExpert in Subplot A
**17-7-18**	Oidium	MILORD	500 cc/ha	
**8-8-18**	Mildew	CALDO BORDELÉS RSR DISPERS	3 kg/ha	
**25-8-18**	Mildew	CALDO BORDELÉS RSR DISPERS	3 kg/ha	Mildew in Greetnal and Basofoliar in Subplot B.
**27-8-18**	Mildew	CUPROXAT 34.5	0.8 %	-*Botrytis cinerea* in Basofoliar in subplot A.<br>-Powdery mildew in SoilExpert in Subplot B.
**5-3-19**	Grass	ROUNDUP PLUS		
**3-5-19**	Dust Mites	EVIPOR	100 cc/ha	-Yesca desease in Control and Greetnal in subplot A.<br>-Condition of *Empoasca* spp. in Greetnal of Subplot A.
**6-5-19**	Oidium	MIOROTHIOL SPECIAL DISPERS	2 kg/ha	
**11-5-19**	Erinosis	EVIPOR	100 cc/ha	-Condition in SoilExpert in Subplot A.
**13-5-19**	Iron corector	HUMITEC EXTRA DRY	3 kg/ha	
**29-5-19**	Mildew	CAPRIF	2 kg/ha	
**29-5-19**	Oidium	ATTENZO STAR	200 cc/ha	
**17-6-19**	Mildew	MELODI CONBI	1.5 kg/ha	
**17-6-19**	Oidium	LUNA EXPERIENCE	400 cc/ha	
**12-7-19**	Mildew	MILRAD-PRO	400 cc/ha	
**12-7-19**	Oidium	PROSPER EC	500 cc/ha	
**13-8-19**	Mildew	CUPRITAL SUPER	2 kg/ha	-Hail damage in SoilExpert in Subplot A.

Table S2. Content of carbohydrates (g Kg^{-1}), free AAs (mg Kg^{-1}), and AcOrg [g Kg^{-1}, except fumaric acid (mg Kg^{-1})] of *Vitis vinifera* leaves after first and 24 hours before first application (T0A and T24B), after and 24 hours before the second foliar application (T0B and T24B) and maturity (TF) in two subplots (A and B) of *Vitis vinifera* untreated (Control) or treated with BABA or three commercial biostimulants [Greetnal (GT), Basofoliar (BF), or SoilExpert (ST)] grown in 2018 and 2019. Average ± standard error.

Treatment	Time	Carbohydrates				Free amino acids							Organic acids						
		**Fruct**	**Gluc**	**Sac**	**Malt**	**Asp**	**Glu**	**Gln**	**Arg**	**Ala**	**GABA**	**BABA**	**Oxal**	**Tart**	**Mal**	**Acet**	**Cit**	**Suc**	**Fum**
2018 Subplot A																			
**C**	**T0A**	40±0.3	53.1±1.3	<LOQ	106.2±2.1	75.6±2.7	91.5±0.1	290.4±3.6	19.6±0.4	89.1±1.7	88.4±1.6	10.2±0.0	1.4±0.0	4.8±0.0	3.8±0.0	1.1±0.0	0.6±0.0	0.3±0.0	2.2±0.0
	**T24A**	17.2±0.2	23.5±0.3	<LOQ	29.0±0.7	81.5±2.4	140.7±2.1	764.8±20.5	20.9±0.2	101.7±0.8	138.3±5.5	9.1±0.2	2.1±0.0	5.8±0.0	4.6±0.0	0.7±0.0	0.6±0.0	0.3±0.0	4.2±0.0
	**T0B**	17.5±0.2	27.9±0.3	<LOQ	31.5±0.5	77.4±2.2	169.4±1.5	113.2±0.2	<LOQ	52.3±1.6	63.4±0.5	8.2±0.0	1.3±0.0	7.8±0.1	5.7±0.0	1.8±0.0	0.2±0.0	0.3±0.0	2.2±0.0
	**T24B**	19.3±0.5	39.5±0.3	<LOQ	489.1±11	52.3±2.3	79.3±0.1	182.6±7.4	<LOQ	55.9±2.0	42.1±1.6	10.0±0.3	1.2±0.0	7.7±0.0	2.7±0.0	6.4±0.1	0.1±0.0	0.2±0.0	1.5±0.0
	**TF**	11.0±0.1	18.0±0.1	<LOQ	11.9±0.1	8.4±0.1	18.8±0.6	8.1±0.1	<LOQ	14.7±0.1	<LOQ	<LOQ	0.7±0.0	2.1±0.0	4.6±0.0	0.4±0.0	0.4±0.0	0.1±0.0	2.3±0.0
**BABA**	**T0A**	25.1±0.5	44.7±0.5	<LOQ	80.0±2.2	90.5±2.7	97.4±4.2	497.7±5.2	28.2±0.3	78.9±3.0	64.2±0.6	9.8±0.0	2.3±0.0	4.6±0.0	4.2±0.0	0.4±0.0	0.2±0.0	0.2±0.0	2.7±0.0
	**T24A**	14.2±0.0	19.4±0.1	<LOQ	30.7±0.7	75.8±2.0	112.1±1.2	1236.6±44.3	28.1±1.2	93.5±0.1	99±0.1	7.5±0.0	2.9±0.0	5.1±0.0	4.2±0.0	0.4±0.0	0.2±0.0	0.2±0.0	4.2±0.0
	**T0B**	20.3±0.4	28.5±0.5	<LOQ	44.5±1.0	78.5±0.9	224.4±7.5	1142.4±8.5	17.8±0.1	105.5±0.3	148.7±4.8	<LOQ	1.3±0.0	5.9±0.0	3.9±0.0	0.6±0.0	0.2±0.0	0.2±0.0	1.6±0.0
	**T24B**	19.6±0.2	25.3±0.5	<LOQ	48.7±0.8	73.9±2.9	76.0±1.5	315.2±5.9	0.1±0.0	45.1±1.1	22.7±0.4	<LOQ	1.7±0.0	5.7±0.0	4.8±0.0	1.1±0.0	0.2±0.0	0.3±0.0	1.8±0.0
	**TF**	15.6±0.3	18.6±0.3	<LOQ	<LOQ	1.3±0.2	27.3±0.4	12.0±0.0	<LOQ	17.7±0.0	<LOQ	<LOQ	1.2±0.0	2.3±0.0	7.5±0.0	0.6±0.0	0.7±0.0	0.2±0.0	1.6±0.0
**GT**	**T0A**	15.2±0.2	20.5±0.3	<LOQ	32.3±0.7	95.0±3.2	82.3±1.0	322.8±6.2	29.5±1.5	81.6±2.4	84.8±0.2	9.3±0.1	1.7±0.0	4.2±0.0	2.7±0.0	0.8±0.0	0.3±0.0	0.1±0.0	2.2±0.0
	**T24A**	15.0±0.3	20.5±0.4	<LOQ	22.7±0.5	83.0±2.6	125±0.7	1191.8±5.3	33.1±0.8	109.5±0.9	169.7±2.4	9.8±0.0	2.5±0.0	4.5±0.0	4.0±0.0	0.9±0.0	0.3±0.0	0.2±0.0	4.4±0.1
	**T0B**	11.0±0.2	23.3±0.5	<LOQ	22.2±0.3	15.1±0.2	48.8±0.8	100.1±2.5	<LOQ	19.0±0.3	34.5±0.7	3.2±0.0	2.0±0.0	7.0±0.1	4.5±0.1	1.4±0.0	0.3±0.0	0.2±0.0	1.5±0.0
	**T24B**	12.8±0.2	20.1±0.4	<LOQ	36.2±0.9	44.4±1.5	75.4±1.4	137.0±3.7	<LOQ	50.9±1.3	<LOQ	7.7±0.0	2.1±0.0	6.5±0.0	4.4±0.0	1.5±0.0	0.3±0.0	0.2±0.0	1.5±0.0
	**TF**	19.1±0.2	21.3±0.4	<LOQ	31.3±0.1	5.5±0.1	27.3±0.1	46.4±0.9	<LOQ	16.0±0.3	<LOQ	<LOQ	2.7±0.0	3.8±0.0	6.5±0.0	0.8±0.0	0.7±0.0	0.3±0.0	1.9±0.0
**BF**	**T0A**	11.9±0.2	16.7±0.1	<LOQ	20.8±0.3	71.0±2.6	89.2±1.6	290.6±3.9	20.9±0.5	129.5±2.5	247.2±2.0	10.8±0.0	1.5±0.0	3.8±0.0	2.6±0.0	0.7±0.0	0.3±0.0	0.2±0.0	2.0±0.0
	**T24A**	11.4±0.3	15.7±0.1	<LOQ	18.2±0.4	39.0±0.7	90.6±1.6	591.4±16.9	<LOQ	100.9±1.3	126.3±1.3	<LOQ	1.9±0.0	5.3±0.0	3.5±0.0	0.7±0.0	0.3±0.0	0.2±0.0	3.0±0.0

	**T0B**	17.3±0.2	24.0±0.4	<LOQ	35.1±0.5	49.8±1.3	172.2±4.8	67.6±2.1	<LOQ	36.2±0.9	65.3±2.5	3.8±0.1	1.4±0.0	5.8±0.0	2.6±0.0	0.8±0.0	0.3±0.0	0.2±0.0	1.0±0.0
	**T24B**	15.6±0.3	30.6±0.4	<LOQ	41.5±0.8	67.9±1.2	75.0±2.8	133.5±1.7	<LOQ	61.2±1.0	<LOQ	4.9±0.0	1.4±0.0	6.7±0.0	4.5±0.0	0.9±0.0	0.3±0.0	0.2±0.0	1.4±0.0
	**TF**	31.6±0.6	11.3±0.2	<LOQ	<LOQ	<LOQ	24.7±0.9	42.8±0.3	<LOQ	8.9±0.2	<LOQ	<LOQ	1.8±0.0	5.7±0.0	5.0±0.0	0.6±0.0	0.8±0.0	0.5±0.0	1.3±0.0
**ST**	**T0A**	10.6±0.1	15.7±0.4	<LOQ	22.8±0.5	56.4±1.9	81.4±0.2	228.1±3.8	15.9±0.2	50.5±0.1	48.1±1.7	8.4±0.2	2.6±0.0	5.5±0.0	3.0±0.0	1.4±0.0	0.4±0.0	0.2±0.0	3.8±0.0
	**T24A**	12.5±0.3	17.1±0.3	<LOQ	17.7±0.5	38.8±0.5	53.3±0.7	433.7±11.9	16.3±0.0	58.7±2.5	84.3±3.3	8.0±0.2	2.7±0.0	5.9±0.0	3.9±0.0	0.9±0.0	0.3±0.0	0.1±0.0	3.6±0.1
	**T0B**	47.4±1.2	29.4±0.7	<LOQ	124.4±3.5	59±1.1	155.5±2.1	87.7±1.7	<LOQ	60.3±1.0	100.8±0.2	6.3±0.0	1.3±0.0	8.6±0.1	3.2±0.1	2.1±0.1	0.1±0.0	0.2±0.0	1.5±0.0
	**T24B**	20.5±0.3	21.8±0.1	<LOQ	42.2±0.7	68.3±2.3	45.2±1.4	118.7±5.7	<LOQ	48.8±1.5	<LOQ	5.1±0.1	1.2±0.1	8.0±0.1	4.0±0.0	1.8±0.0	0.1±0.0	0.3±0.0	1.9±0.0
	**TF**	7.1±0.1	11.4±0.1	<LOQ	<LOQ	5.0±0.0	28.1±0.2	63.8±2.6	<LOQ	12.8±0.3	<LOQ	<LOQ	1.2±0.0	2.1±0.0	6.2±0.0	0.3±0.0	0.3±0.0	0.2±0.0	3.7±0.0
2018 Subplot B																		
**C**	**T0A**	9.1±0.1	11.8±0.2	<LOQ	24.1±0.7	51.8±0.4	72.7±2.0	202.2±5.8	13.6±0.2	55.8±0.0	80.9±2.1	8.5±0.1	1.8±0.0	5.4±0.0	3.3±0.0	1.3±0.0	0.7±0.0	0.2±0.0	2.7±0.0
	**T24A**	10.3±0.2	10.8±0.1	<LOQ	7.8±0.1	56.8±0.5	94.9±1.5	726.9±34.4	<LOQ	106.7±0.5	132.7±3.9	<LOQ	2.0±0.0	5.6±0.0	4.7±0.0	1.7±0.0	1.3±0.0	0.7±0.0	5.0±0.0
	**T0B**	11.6±0.2	21.4±0.6	<LOQ	28.1±0.2	54.8±2.7	222.8±4.9	232.0±6.0	15.2±0.0	65.9±1.0	69.3±1.6	<LOQ	1.8±0.0	6.6±0.0	3.9±0.0	5.4±0.0	0.2±0.0	0.2±0.0	1.5±0.0
	**T24B**	13.8±0.1	20.2±0.1	<LOQ	38.5±0.8	58.6±0.9	76.3±2.9	127.6±1.1	<LOQ	48.1±0.6	<LOQ	<LOQ	1.9±0.0	6.9±0.0	5.0±0.0	3.4±0.0	0.2±0.0	0.4±0.0	1.7±0.0
	**TF**	14.5±0.2	16.6±0.2	<LOQ	25.0±0.6	3.3±0.0	29.9±1.4	13.4±0.7	<LOQ	15.3±0.7	<LOQ	<LOQ	1.0±0.0	2.1±0.0	8.0±0.0	0.4±0.0	0.6±0.0	0.1±0.0	1.7±0.0
**BABA**	**T0A**	5.6±0.2	1.5±0.0	<LOQ	21.6±0.5	102±4.6	52.6±0.1	241.6±3.8	29.0±0.4	74.2±3.3	82.2±2.9	13.1±0.0	2.1±0.0	4.5±0.0	3.0±0.0	0.9±0.0	0.4±0.0	0.1±0.0	2.6±0.0
	**T24A**	6.8±0.2	8.7±0.2	<LOQ	<LOQ	29.1±0.2	124.8±1.7	663.0±2.4	<LOQ	91.3±3.3	81.7±4.1	12.6±0.1	3.1±0.1	4.9±0.0	4.9±0.0	1.3±0.0	0.4±0.0	0.5±0.0	4.0±0.0
	**T0B**	15.0±0.5	17.0±0.6	<LOQ	26.7±0.4	58.2±0.8	159.8±8.1	156.3±3.8	<LOQ	96.6±2.2	124.7±0.1	<LOQ	2.3±0.0	5.8±0.0	3.6±0.0	1.8±0.0	0.3±0.0	0.4±0.0	1.4±0.0
	**T24B**	12.9±0.0	16.6±0.2	<LOQ	41.6±0.8	54.8±2.6	56.6±1.0	<LOQ	<LOQ	69.2±2.4	3.7±0.0	<LOQ	2.0±0.0	6.2±0.0	3.7±0.0	1.7±0.0	0.3±0.0	0.6±0.0	2.0±0.0
	**TF**	16.1±0.7	19.8±0.3	<LOQ	29.7±1.0	5.1±0.3	61.4±0.9	2.6±0.1	<LOQ	23.5±0.3	<LOQ	<LOQ	2.2±0.0	4.2±0.0	6.3±0.0	0.6±0.0	0.7±0.0	0.1±0.0	1.7±0.0
**GT**	**T0A**	20.6±0.6	21.8±0.8	<LOQ	27.4±0.2	123.5±3.3	156.4±5.3	602.5±2.7	41.4±1.0	111.0±2.5	106.4±2.3	10.4±0.2	2.0±0.0	4.8±0.0	3.9±0.0	0.9±0.0	0.2±0.0	0.2±0.0	2.4±0.0
	**T24A**	10.2±0.3	11.8±0.2	<LOQ	14.2±0.2	29.4±0.1	73.1±2.3	531.3±12.5	<LOQ	85.3±2.6	84.8±3.6	<LOQ	2.8±0.0	5.0±0.0	3.7±0.0	1.0±0.0	0.3±0.0	0.2±0.0	5.2±0.0
	**T0B**	15.0±0.1	21.5±0.2	<LOQ	22.0±0.7	54.7±1.7	127.4±1.7	112.7±3.3	<LOQ	64.1±0.6	80.7±0.7	<LOQ	1.8±0.0	5.8±0.0	6.0±0.0	0.9±0.0	0.4±0.0	0.1±0.0	1.5±0.0

	**T24B**	13.1±0.5	22.3±1.0	<LOQ	41.9±2.1	31.3±1.1	49.7±1.4	282.6±5.4	<LOQ	36.5±0.8	<LOQ	<LOQ	2.0±0.0	4.9±0.0	5.4±0.0	0.8±0.0	0.4±0.0	0.2±0.0	1.6±0.0
	**TF**	13.9±0.3	13.3±0.1	<LOQ	23.2±0.3	9.1±0.2	34.8±0.1	34.4±1.6	<LOQ	23.4±0.4	<LOQ	<LOQ	3.0±0.0	2.2±0.0	9.2±0.0	0.6±0.0	0.9±0.0	0.2±0.0	2.7±0.0
**BF**	**T0A**	10.4±0.1	9.6±0.2	<LOQ	28.9±0.6	92.8±4.8	73.5±2.7	343.3±2.4	27.2±1.2	67.5±2.6	99.8±4.2	10.5±0.2	2.1±0.0	4.8±0.0	3.8±0.0	0.6±0.0	0.4±0.0	0.2±0.0	3.3±0.0
	**T24A**	8.5±0.4	9.6±0.2	<LOQ	6.8±0.3	36.7±1.4	88.6±4.2	647.0±35.5	<LOQ	114.6±1.6	105.4±5.4	<LOQ	2.5±0.0	4.8±0.0	3.8±0.0	0.8±0.0	0.4±0.0	0.2±0.0	4.9±0.0
	**T0B**	17.2±0.8	21.9±0.9	<LOQ	36.0±1.5	33.3±1.7	148.6±6.8	155.3±5.7	<LOQ	40.6±1.5	85.0±3.5	1.1±0	2.9±0.0	5.3±0.0	5.8±0.0	1.4±0.0	0.4±0.0	0.7±0.0	2.0±0.0
	**T24B**	10.0±0.5	15.7±0.6	<LOQ	53.1±2.5	25.3±0.8	42.7±0.6	282.7±0.7	<LOQ	37.0±1.7	<LOQ	<LOQ	2.3±0.0	6.5±0.0	3.8±0.0	1.0±0.0	0.4±0.0	0.4±0.0	1.9±0.0
	**TF**	14.7±0.5	13.2±0.4	<LOQ	<LOQ	<LOQ	27.0±0.5	1.3±0.0	<LOQ	16.4±0.3	<LOQ	<LOQ	1.3±0.0	2.8±0.0	6.4±0.0	0.5±0.0	0.8±0.0	0.3±0.0	1.9±0.0
**ST**	**T0A**	12.0±0.1	17.3±0.4	<LOQ	27.1±0.2	113.0±3.0	114.4±3.4	337.8±7	29.0±0.5	98.1±1.9	92.5±2.8	12.3±0.4	2.8±0.0	4.5±0.0	3.0±0.0	0.8±0.0	0.3±0.0	0.1±0.0	4.5±0.0
	**T24A**	9.1±0.1	12.1±0.2	<LOQ	<LOQ	51.4±1.3	111.6±0.9	660.6±21.6	<LOQ	157.0±6.3	137.5±6.6	1.1±0	2.8±0.0	5.0±0.0	3.9±0.0	0.7±0.0	0.1±0.0	0.1±0.0	7.7±0.1
	**T0B**	14.1±0.2	17.7±0.2	<LOQ	22.1±0.1	23.3±0.4	120.1±4.6	46.3±1.8	<LOQ	49.1±0.3	43.6±1.0	1.1±0	1.8±0.0	6.1±0.1	7.6±0.0	0.9±0.0	0.3±0.0	0.2±0.0	2.0±0.0
	**T24B**	14.0±0.3	28.0±0.6	<LOQ	37.0±0.5	51.8±0.6	91.9±0.8	314.3±7.5	<LOQ	65.2±0.8	<LOQ	<LOQ	1.5±0.0	6.1±0.0	5.0±0.0	1.1±0.0	0.2±0.0	0.3±0.0	2.0±0.0
	**TF**	13.3±0.4	17.0±0.2	<LOQ	18.5±0.5	14.5±0.5	18.4±0.5	2.6±0.0	<LOQ	12.9±0.4	<LOQ	<LOQ	0.8±0.0	2.1±0.0	3.4±0.0	0.4±0.0	0.2±0.0	0.1±0.0	3.2±0.0
2019 Subplot A																		
**C**	**T0A**	19.3±1.0	23.3±0.9	1.8±0.1	25.6±1.0	18.4±0.2	51.8±1.4	317.1±6.6	7.3±0.2	106.6±4.5	415.9±14.0	41.1±0.6	0.9±0.0	7.6±0.2	0.1±0.0	2.7±0.1	<LOQ	0.1±0.0	2.8±0.1
	**T24A**	19.3±0.3	35.2±0.4	<LOQ	41.2±0.9	17.8±0.7	46.3±0.5	234.4±2.0	12.8±0.0	186.1±1.1	334.2±3.5	31.2±1.1	0.8±0.0	5.9±0.6	<LOQ	6.4±0.3	0.3±0.0	0.4±0.0	2.6±0.3
	**T0B**	16.3±0.2	17.4±0.4	<LOQ	39.5±1.1	9.0±0.1	18.0±0.3	96.4±3.7	9±0.1	30.1±0.6	50.6±1.2	7±0	0.8±0.0	7.2±0.0	0.1±0.0	5.7±0.0	0.1±0.0	0.4±0.0	1.7±0.0
	**T24B**	20.1±0.6	24.1±0.5	<LOQ	28.4±0.4	22.6±0.6	29.5±0.5	40.6±1.0	2.0±0.0	39.5±1.1	29.9±0.2	1.6±0.0	0.7±0.0	4.3±0.2	0.1±0.0	1.9±0.0	0.2±0.0	0.4±0.0	1.9±0.1
	**TF**	21.0±1.0	20.8±0.7	8.2±0.3	20.5±0.6	72.5±1.0	32.6±0.1	48.5±0.7	24.2±0.4	23.2±0.2	75.3±1.0	1.6±0.0	0.6±0.0	3.7±0.0	0.1±0.0	0.8±0.1	0.6±0.0	0.6±0.0	5.0±0.2
**BABA**	**T0A**	16.5±0.7	20.2±0.8	<LOQ	27.8±0.9	23±0.4	89.4±1.4	1046.5±0.7	30.3±0.1	68.1±1.7	192.0±0.5	20.3±0.1	1.5±0.0	6.4±0.0	0.1±0.0	3.5±0.0	0.1±0.0	0.6±0.0	4.1±0.2
	**T24A**	18.1±0.5	27.1±1.3	<LOQ	22.4±0.9	20.1±0.3	39.1±1.5	256.5±4.7	17.1±0.1	234.4±1.8	337.3±0.4	34.8±0.3	0.9±0.1	6.0±0.0	<LOQ	5.0±0.1	0.3±0.0	0.3±0.0	3.7±0.1
	**T0B**	17.2±0.7	29.5±1.0	<LOQ	40.2±1.2	25.8±1.0	49.1±1.6	47.1±1.4	12.4±0.3	49.4±1.7	35.3±0.9	5.2±0.1	0.6±0.0	6.3±0.6	0.1±0.0	3.9±0.1	0.2±0.0	0.3±0.0	1.7±0.1
	**T24B**	18.6±0.8	29.9±1.3	<LOQ	37.9±1.6	28.2±0.3	43.4±0.6	10.4±0.2	9.0±0.0	49.9±0.9	48.7±1.2	2.5±0.0	0.7±0.0	5.8±0.0	0.1±0.0	2.8±0.1	0.2±0.0	0.4±0.0	2.4±0.0

	**TF**	19.7±0.9	17.8±0.7	5.1±0.2	17.9±0.3	81.9±1.3	38.6±0.7	91.2±0.3	27.0±0.1	30.3±0.3	99.4±0.4	3.0±0.0	0.5±0.0	4.0±0.2	0.1±0.0	0.8±0.0	0.6±0.0	0.6±0.0	3.2±0.0
	**T0A**	15.3±0.0	18.5±0.1	<LOQ	10.9±0.5	32.0±0.7	100.5±1.2	829.7±2.5	17.7±0.1	173.2±3.9	380.8±1.6	38.4±0.2	1.1±0.0	5.4±0.1	0.1±0.0	2.3±0.1	0.1±0.0	0.3±0.0	4.9±0.1
	**T24A**	19.7±0.7	28.4±1.4	<LOQ	35.2±0.5	29.0±0.5	118.5±0.4	476.9±15.5	23.6±0.3	210.9±2.5	353.1±5.8	36.8±0.8	0.7±0.0	5.5±0.4	<LOQ	5.5±0.4	0.3±0.0	0.2±0.0	2.1±0.2
**GT**	**T0B**	22.0±1.2	28.2±1.0	<LOQ	53.3±2.4	26.7±0.1	52.3±1.7	140.5±2.2	24.8±0.6	80.6±1.4	92.1±0.4	10.4±0.0	0.8±0.0	6.5±0.1	0.1±0.0	5.2±0.2	0.1±0.0	0.2±0.0	1.5±0.1
	**T24B**	11.5±0.1	21.0±0.8	<LOQ	33.5±1.2	33.7±0.7	49.6±0.9	5.7±0.1	6.4±0.0	55.2±0.7	30.6±0.6	2.4±0.0	0.8±0.0	5.6±0.0	0.1±0.0	2.6±0.0	0.5±0.0	0.4±0.0	3.5±0.0
	**TF**	22.5±0.9	25.6±0.2	7.7±0.2	18.7±1.1	100.1±0.3	42.4±1.2	107.8±0.4	32.2±0.5	41.3±1.3	132.6±3.0	4.6±0.0	0.6±0.0	4.5±0.1	0.1±0.0	0.7±0.1	0.4±0.0	0.8±0.0	3.2±0.2
	**T0A**	14.3±0.4	17.3±0.5	<LOQ	<LOQ	33.9±0.6	111.2±3.7	414.2±0.3	8.6±0.0	132.1±1.2	281.5±5.5	28.4±0.2	0.7±0.0	5.9±0.2	0.1±0.0	3.4±0.0	0.1±0.0	0.2±0.0	4.9±0.4
	**T24A**	19.6±0.9	27.8±0.5	<LOQ	19.1±0.1	15.5±0.3	59.7±0.3	155.3±3.4	9.1±0.3	119.6±0.3	363.4±5.5	33.8±0.1	0.8±0.0	6.7±0.5	0.1±0.0	4.4±0.3	0.3±0.0	0.4±0.0	2.8±0.1
**BF**	**T0B**	20.2±0.6	32.2±1.5	<LOQ	28.1±1.1	21.6±0.6	33.3±0.6	30.7±0.4	8.5±0.2	43.8±0.2	30.8±0.4	5.3±0.1	0.7±0.0	7.7±0.4	0.1±0.0	4.0±0.0	0.2±0.0	0.3±0.0	1.7±0.0
	**T24B**	14.7±0.7	29.3±1.0	<LOQ	29.4±0.3	43.7±0.5	100.1±0.4	62.7±1.4	9.7±0.3	114.1±1.6	65.5±1.6	2.4±0.0	0.7±0.0	7.5±0.1	0.1±0.0	2.8±0.0	0.1±0.0	0.4±0.0	1.4±0.0
	**TF**	6.6±0.0	9.0±0.4	2.3±0.1	14.7±0.3	108.8±3.4	48.6±0.3	38.0±0.6	41.1±1.8	36.1±1.0	109.8±2.0	2.7±0.0	0.5±0.0	4.5±0.0	0.1±0.0	0.6±0.0	0.5±0.0	0.6±0.0	4.8±0.0
	**T0A**	19.8±0.6	20.9±0.5	<LOQ	11.4±0.5	36.3±0.3	90.7±1.1	893.5±27.4	14.1±0.7	98.2±4.6	230.9±6.3	21.9±0.0	0.8±0.1	6.7±0.3	0.1±0.0	3.6±0.4	<LOQ	0.3±0.0	4.2±1.5
	**T24A**	13.2±0.6	18.7±1.0	<LOQ	13.7±0.7	10.4±0.4	23.8±0.6	226.8±4.4	10.3±0.4	126.6±2.0	188.6±1.9	17.3±0.2	0.9±0.0	6.7±0.1	<LOQ	6.3±0.0	0.3±0.0	0.6±0.0	2.1±0.0
**ST**	**T0B**	16.7±0.6	26.6±1.0	<LOQ	39.1±0.4	18.6±0.0	45.6±1.0	98.5±2.8	10.2±0.1	45.3±0.8	52.3±1.1	7.8±0.0	0.6±0.0	5.9±0.4	0.1±0.0	5.2±0.0	0.2±0.0	0.5±0.0	1.2±0.1
	**T24B**	20.8±0.7	33.4±1.4	<LOQ	28.4±0.3	43.4±0.1	111.4±0.5	67.5±0.3	10.0±0.3	95.5±1.5	49.0±0.7	2.9±0.0	0.7±0.0	6.1±0.2	0.1±0.0	4.2±0.1	0.3±0.0	0.5±0.0	1.6±0.0
	**TF**	16.8±0.5	19.0±0.4	7.7±0.1	18.0±0.2	77.4±0.3	35.6±0.4	55.0±1.4	26.9±0.5	25.6±0.3	83.3±0.3	2.1±0.0	0.5±0.0	4.3±0.3	<LOQ	0.6±0.0	0.4±0.0	0.6±0.0	3.9±0.1
2019 Subplot B																			
	**T0A**	19.5±0.7	34.5±0.4	<LOQ	25.1±0.5	29.0±0.6	74.7±3.0	317.7±9.5	10.4±0.2	126.2±2.9	268.6±3.8	26.7±0.6	1.0±0.1	6.8±0.0	0.1±0.0	1.0±0.1	1.4±0.1	0.1±0.0	6.3±0.2
	**T24A**	23.7±1.3	30.9±0.1	<LOQ	21.7±0.3	22.8±0.2	88.5±1.4	245.6±2.4	10.0±0.2	59.2±0.6	140.0±3.5	15.4±0.2	0.4±0.0	6.2±0.1	<LOQ	0.8±0.0	1.4±0.0	0.4±0.0	1.7±0.1
**C**	**T0B**	10.9±0.5	17.8±0.8	<LOQ	22.7±0.8	16.3±0.1	26.8±0.3	85.5±1.1	11.3±0.1	50.6±0.3	88.7±0.4	10.9±0.0	0.7±0.0	7.3±0.3	0.1±0.0	0.6±0.0	0.7±0.0	0.3±0.0	3.0±0.1
	**T24B**	11.9±0.2	17.8±0.6	<LOQ	17.7±0.6	41.4±1.3	47.2±0.7	54.8±0.3	9.3±0.2	64.2±1.0	44.5±1.2	2.2±0.0	1.0±0.0	8.3±0.2	0.1±0.0	1.0±0.0	1.6±0.0	0.2±0.0	4.1±0.1
	**TF**	16.0±0.7	17.4±0.6	5.8±0.2	28.5±1.0	71.5±1.4	31.4±0.4	34.5±0.7	25.7±0.5	21.6±0.6	81.0±0.2	1.3±0.0	0.7±0.0	3.3±0.0	0.1±0.0	0.3±0.0	0.8±0.0	0.3±0.0	4.6±0.1

**BABA**	**T0A**	21.7±0.3	28.8±1.1	<LOQ	18.5±0.2	31.3±0.7	61.9±0.2	774.4±14.4	29.7±0.7	108.0±3.5	238.6±10.4	23.6±0.7	0.9±0.0	3.7±0.0	0.1±0.0	0.3±0.0	2.7±0.2	0.4±0.0	5.8±0.0
	**T24A**	23.9±1.2	35.5±1.8	<LOQ	32.1±0.1	32.5±0.2	90.1±0.3	469.7±8.9	17.8±0.1	121.2±0.2	323.5±5.4	31.6±0.5	1.0±0.1	5.9±0.0	<LOQ	0.3±0.0	1.4±0.0	0.4±0.0	2.0±0.0
	**T0B**	14.3±0.4	25.4±1.2	<LOQ	28.3±0.5	26.5±0.2	79.4±2.3	92.8±0.7	17.7±0.3	103.5±2.4	93.7±0.8	13.1±0.1	0.8±0.0	6.9±0.1	0.1±0.0	0.6±0.0	0.8±0.0	0.3±0.0	2.5±0.1
	**T24B**	16.5±0.9	28.4±1.2	<LOQ	53.4±2.6	62.3±2.0	78.0±3.1	103.5±1.0	15.6±0.5	137.0±6.0	104.8±3.9	2.8±0.0	0.8±0.0	5.8±0.2	0.1±0.0	0.4±0.0	0.9±0.0	0.3±0.0	2.0±0.1
	**TF**	13.9±0.2	17.8±0.3	9.9±0.3	20.0±0.9	95.3±0.4	39.2±0.2	33.3±0.6	34.8±0.4	34.6±1.0	86.6±0.8	1.0±0.0	0.9±0.0	3.6±0.1	0.1±0.0	0.4±0.0	0.9±0.0	0.5±0.0	5.8±0.2
**GT**	**T0A**	22.8±0.9	36.7±1.4	<LOQ	34.4±1.5	34.1±0.7	88.5±0.3	331.8±2.9	0.9±0.0	55.2±1.3	101.3±2.8	11.9±0.2	2.1±0.0	5±0.1.0	0.1±0.0	0.1±0.0	0.6±0.0	0.1±0.0	5.5±0.0
	**T24A**	20.4±0.4	33.3±0.0	<LOQ	33.9±1.1	15.7±0.4	23.2±0.2	170.0±5.7	7.3±0.1	219.0±1.8	275.9±5.9	26.0±0.3	0.7±0.0	3.9±0.0	<LOQ	0.5±0.0	2.1±0.0	0.6±0.1	2.7±0.2
	**T0B**	9.1±0.3	13.4±0.4	<LOQ	10.8±0.5	21.8±0.6	32.1±0.8	18.1±0.5	16.2±0.4	58.3±1.8	42.2±0.6	5.6±0.1	0.9±0.0	6.3±0.2	0.1±0.0	0.9±0.0	0.9±0.0	0.2±0.0	2.8±0.1
	**T24B**	16.3±0.7	30.0±1.2	<LOQ	45.7±1.5	72.2±1.9	103.4±2.9	62.7±1.2	10.9±0.0	121.7±2.8	47.7±1.0	3.4±0.0	0.9±0.1	6.6±0.2	0.1±0.0	1.2±0.0	2.2±0.0	0.2±0.0	2.5±0.1
	**TF**	13.7±0.5	18.9±0.6	2±0.1	16.7±0.6	76.8±2.3	30.7±0.6	50.6±0.4	26.9±0.5	20.8±0.6	62.2±1.0	0.2±0.0	1.4±0.0	3.9±0.0	0.1±0.0	0.6±0.0	1.5±0.0	0.3±0.0	9.5±0.0
**BF**	**T0A**	18.9±0.9	32.3±1.6	<LOQ	21.7±0.1	22.3±0.6	50.7±0.7	605.4±23.4	29.5±1.0	78.6±1.7	195.4±3.5	20.4±0.4	0.5±0.0	5.1±0.4	0.1±0.0	0.4±0.0	1.8±0.1	0.3±0.0	3.4±0.3
	**T24A**	18.1±0.5	33.0±0.4	<LOQ	32.9±0.9	28.1±0.5	136.3±4.8	299.3±1.3	14.5±0.3	184.4±0.2	339.4±5.8	33.7±0.7	0.9±0.1	4.9±0.1	<LOQ	0.5±0.0	2.2±0.1	0.5±0.0	2.4±0.0
	**T0B**	12.1±0.6	23.1±0.9	<LOQ	38.3±1.9	20.8±0.4	42.0±0.8	58.9±1.8	12.8±0.1	50.8±1.6	67.7±2.1	7.0±0.1	0.8±0.1	5.9±0.1	0.1±0.0	0.8±0.1	0.8±0.1	0.4±0.0	3.2±0.0
	**T24B**	18.7±0.1	28.7±1.5	<LOQ	39.4±1.7	53.0±1.3	87.2±1.8	43.8±1.0	12.2±0.3	96.7±3.6	35.0±0.9	2.9±0.0	1.1±0.0	5.7±0.1	0.1±0.0	0.5±0.0	0.9±0.0	0.3±0.0	3.2±0.3
	**TF**	10.9±0.6	8.4±0.2	<LOQ	6.9±0.2	84.0±0.8	41.2±1.0	94.8±2.4	27.1±0.4	27.5±0.2	99.8±0.4	1.9±0.0	1.1±0.0	3.1±0.0	0.1±0.0	0.4±0.0	1.1±0.0	0.7±0.0	4.6±0.1
**ST**	**T0A**	20.7±0.3	28.7±0.1	<LOQ	19.8±0.7	25.4±0.0	27.8±0.2	226.3±4.0	6.0±0.1	31.9±0.1	86.3±0.3	12.0±0.2	1.6±0.1	5.2±0.3	<LOQ	0.8±0.0	0.8±0.0	0.1±0.0	4.9±0.1
	**T24A**	24.1±0.2	33.9±0.8	<LOQ	34.8±1.6	21.0±0.5	84.3±3.9	256.5±7.4	19.5±0.6	180.3±3.2	410.2±11.2	34.0±0.4	1.0±0.0	6.6±0.3	<LOQ	0.4±0.0	0.9±0.0	0.2±0.0	1.7±0.0
	**T0B**	13.9±0.6	18.6±0.8	<LOQ	22.8±1.0	18.0±0.1	27.1±0.3	13.1±0.4	17.3±0.2	29.1±0.2	32.2±0.9	4.4±0.0	0.9±0.1	3.9±0.1	0.1±0.0	0.2±0.0	0.5±0.0	0.4±0.0	3.7±0.1
	**T24B**	10.2±0.4	21.1±0.8	<LOQ	22.1±0.6	53.0±2.2	63.0±2.0	11.6±0.2	8.2±0.1	84.2±0.8	38.1±1.0	2.3±0.0	0.0.7±0	4.0±0.0	<LOQ	0.4±0.0	0.5±0.0	0.1±0.0	1.9±0.0
	**TF**	15.6±0.2	19.7±0.9	5.6±0.1	28.7±0.9	64.6±0.8	46.4±0.2	56.8±0.5	16.8±0.0	21.6±0.5	100.4±3.9	4.3±0.1	1.0.1±0	1.4±0.0	0.1±0.0	0.6±0.0	1.0±0.0	0.9±0.0	7.4±0.2

Table S3. Content of free AAs (mg Kg-1), carbohydrates (g Kg-1) and AcOrg [g Kg-1, except fumaric acid (mg Kg-1) of Vitis vinifera grapes and carbohydrates (g L-1) free AAs (mg L-1) and AcOrg [g L-1, except fumaric acid (mg L-1)] of Vitis vinifera musts at veraison (TV) and maturity (TF) in two subplots (A and B) of Vitis vinifera untreated (Control) or treated with BABA or three commercial biostimulants [(Greetnal (GT), Basofoliar (BF), or SoilExpert (ST)] grown in 2018 and 2019. Average ± standard Error.

			Carbohydrates		Free amino acids								Orgaic Acids						
	Treatment	Time	Fruct	Gluc	Asp	Glu	Asn	Gln	Arg	Ala	GABA	BABA	Oxal	Tart	Mal	Acet	Cit	Suc	Fum
	Subplot A, 2018																		
Grapes	C	TV	53.8±0.6	57.0±0.4	49.0±1.6	75.3±2.0	<LOQ	25.9±0.9	<LOQ	<LOQ	42.0±0.5	21.8±0.3	0.5±0.0	4.1±0.0	1.5±0.0	0.3±0.0	0.2±0.0	<LOQ	1.7±0.0
		TF	64.3±0.0	72.8±2.7	87.0±2.3	49.3±0.8	<LOQ	37.3±1.4	<LOQ	<LOQ	46.0±0.9	20.4±0.2	1.1±0.0	4.3±0.1	1.1±0.0	0.3±0.0	0.2±0.0	<LOQ	3.0±0.0
	BABA	TV	54.3±0.1	55.8±1.6	68.3±0.1	43.4±1.3	<LOQ	4.0±0.0	<LOQ	<LOQ	24.6±0.6	6.1±0.1	0.2±0.0	6.6±0.0	1.1±0.0	0.2±0.0	0.1±0.0	<LOQ	1.2±0.0
		TF	60.49±0.1	76.8±0.5	94.4±0.1	49.9±0.4	<LOQ	21.0±0.1	<LOQ	<LOQ	26.9±0.1	22.5±0.2	0.3±0.0	4.6±0.0	1.0±0.0	0.2±0.0	0.1±0.0	<LOQ	1.9±0.0
	GT	TV	60.9±1.1	73.4±0.7	63.0±0.6	61.4±0.5	<LOQ	19.5±0.1	<LOQ	<LOQ	27.3±0.4	<LOQ	1.0±0.0	5.6±0.0	1.0±0.0	0.2±0.0	0.2±0.0	<LOQ	2.8±0.0
		TF	58.2±0.6	71.0±0.8	92.5±1.2	18.5±0.3	<LOQ	16.0±0.5	<LOQ	<LOQ	22.9±0.2	13.5±0.2	1.6±0.0	4.8±0.0	0.9±0.0	0.3±0.0	0.2±0.0	<LOQ	3.8±0.0
	BF	TV	52.6±0.1	62.0±0.6	112.3±0.5	54.8±1.1	<LOQ	17.4±0.1	<LOQ	<LOQ	32.5±0.1	3.4±0.0	0.5±0.0	6.2±0.0	1.2±0.0	0.2±0.0	0.5±0.0	<LOQ	1.4±0.0
		TF	56.8±0.6	76.3±0.6	83.3±1.2	25.8±0.6	<LOQ	11.3±0.2	<LOQ	<LOQ	25.4±0.2	14.6±0.1	0.8±0.0	4.2±0.1	1.3±0.0	0.2±0.0	271.9±2.7	<LOQ	2.2±0.0
	ST	TV	40.6±0.3	49.0±0.6	74.3±0.8	77.7±0.3	<LOQ	15.5±0.4	<LOQ	<LOQ	34.9±0.1	27.2±0.5	0.4±0.0	5.9±0.0	1.4±0.0	0.2±0.0	0.2±0.0	<LOQ	1.2±0.0
		TF	74.2±1.3	84.9±1.1	118.7±1.0	61.6±0.9	<LOQ	18.6±0.6	<LOQ	<LOQ	41.1±0.1	15.8±0.3	0.5±0.0	6.6±0.0	1.4±0.0	0.2±0.0	0.2±0.0	<LOQ	3.3±0.0
	Subplot B, 2018																		
	C	TV	57.4±0.8	73.8±1.2	<LOQ	<LOQ	<LOQ	39.2±0.6	111.0±1.5	2.6±0.0	78.2±0.9	3.4±0.0	0.2±0.0	4.8±0.0	1.8±0.0	0.2±0.0	0.1±0.0	<LOQ	1.4±0.0
		TF	59.1±1.0	74.3±1.3	<LOQ	<LOQ	<LOQ	11.7±0.1	<LOQ	<LOQ	28.0±0.2	17.2±0.1	0.4±0.0	3.9±0.0	1.3±0.0	0.2±0.0	0.1±0.0	<LOQ	2.0±0.0
	BABA	TV	78.3±2.1	93.6±2.1	131.6±1.1	18.9±0.1	<LOQ	121.5±2.1	216.3±1.3	27.0±0.0	188.5±2.0	12.5±0.2	0.6±0.0	7.3±0.0	1.8±0.0	0.3±0.0	0.2±0.0	<LOQ	3.1±0.0
		TF	85.6±2.2	103.7±1.6	<LOQ	<LOQ	<LOQ	38.1±0.1	132.2±3.2	<LOQ	107.3±4.3	2.4±0.0	0.7±0.0	6.7±0.0	2.0±0.0	0.6±0.0	0.3±0.0	<LOQ	5.9±0.1
	GT	TV	81.3±0.8	94.9±0.9	98.3±0.9	<LOQ	<LOQ	13.2±0.4	<LOQ	<LOQ	25.2±0.1	<LOQ	0.3±0.0	5.4±0.0	1.7±0.0	0.3±0.0	0.1±0.0	<LOQ	1.9±0.0
		TF	53.0±0.5	66.9±1.1	<LOQ	<LOQ	<LOQ	114.3±2.2	261.6±7.9	27.9±0.5	150.8±0.9	25.9±0.5	0.6±0.0	5.1±0.0	1.1±0.0	0.2±0.0	0.2±0.0	<LOQ	4.0±0.0
	BF	TV	51.8±0.6	56.2±0.7	121.5±2.1	<LOQ	<LOQ	18.3±0.2	<LOQ	<LOQ	39.3±0.8	<LOQ	0.4±0.0	6.4±0.0	1.6±0.0	0.3±0.0	0.3±0.0	0.2±0.0	4.1±0.0

		**TF**	47.9±0.4	58.5±1.6	<LOQ	<LOQ	<LOQ	140.0±2.6	188.1±4.7	19.6±0.3	134.7±4.9	6.0±0.1	0.6±0.0	2.6±0.0	2.2±0.0	0.2±0.0	0.2±0.0	<LOQ	2.8±0.0
	**ST**	**TV**	61.8±1.0	73.0±1.2	<LOQ	<LOQ	<LOQ	43.6±1.8	128.4±1.3	18.4±0.4	82.0±2.6	6.6±0.1	0.3±0.0	5.1±0.0	1.6±0.0	0.3±0.0	0.3±0.0	0.1±0.0	1.7±0.0
		**TF**	<LOQ	<LOQ	<LOQ	<LOQ	<LOQ	68.1±1.6	154.3±3.0	15.8±0.4	108.0±1.9	<LOQ	0.6±0.0	5.0±0.0	1.5±0.0	0.3±0.0	0.4±0.0	0.8±0.0	2.7±0.0
	**Subplot A, 2019**																		
	**C**	**TV**	70.4±1.0	75.1±1.5	75.5±1.0	12.2±0.0	<LOQ	17.5±0.5	174.3±3.9	243.2±5.7	57.4±0.1	82.1±1.0	0.9±0.0	2.4±0.0	3.0±0.0	0.1±0.0	0.2±0.0	0.2±0.0	2.8±0.0
		**TF**	72.8±1.2	78.2±0.5	129.1±2.7	31.1±0.2	<LOQ	14.9±0.3	856.5±15.6	535.7±6.1	112.4±1.3	162.4±2.5	2.1±0.1	3.4±0.0	2.5±0.0	0.1±0.0	0.3±0.0	0.4±0.0	2.8±0.0
	**BABA**	**TV**	37.6±0.9	49.5±0.3	82.1±1.7	24.3±0.4	<LOQ	18.2±0.0	282.9±4.5	407.3±5.4	86.2±3.1	113.1±1.5	1.3±0.0	2.1±0.0	2.3±0.0	0.1±0.0	0.1±0.0	0.1±0.0	2.2±0.0
		**TF**	49.1±0.4	52.2±0.3	89.2±1.8	<LOQ	<LOQ	<LOQ	101.0±0.5	340.2±2.6	32.2±0.8	103.1±2.2	1.2±0.0	2.9±0.0	1.9±0.0	0.1±0.0	0.2±0.0	0.1±0.0	2.0±0.0
	**GT**	**TV**	<LOQ	<LOQ	200.0±2.6	48.4±1.2	<LOQ	31.5±0.2	281.2±6.4	445.7±9.5	110.7±1.3	165.0±3.3	1.1±0.0	3.1±0.0	2.5±0.0	0.1±0.0	0.2±0.0	0.1±0.0	6.5±0.0
		**TF**	80.2±1.0	85.1±1.3	116.4±3.1	<LOQ	<LOQ	0.8±0.0	180.9±5.6	436.0±6.8	66.5±0.1	215.4±5.7	1.3±0.1	2.8±0.0	1.9±0.0	0.1±0.0	0.3±0.0	0.1±0.0	13.1±0.0
	**BF**	**TV**	<LOQ	<LOQ	248.9±3.5	31.5±1.4	<LOQ	15.9±0.1	226.6±3.2	530.0±3.4	93.6±2.1	111.6±0.2	1.1±0.1	2.1±0.0	2.2±0.0	0.1±0.0	0.2±0.0	0.1±0.0	2.3±0.0
		**TF**	87.6±2.0	98.8±0.5	117.5±1.8	<LOQ	<LOQ	5.3±0.0	200.5±0.2	560.7±8.6	64.1±0.7	154.8±1.5	1.5±0.0	2.5±0.0	2.4±0.0	<LOQ	0.2±0.0	0.1±0.0	4.1±0.1
	**ST**	**TV**	73.4±0.1	82.7±2.7	54.4±0.7	10.0±0.2	<LOQ	16.4±0.4	150.7±0.0	254.3±2.2	53.3±0.3	85.1±0.3	1.0±0.0	2.9±0.0	3.2±0.0	0.1±0.0	0.3±0.0	0.2±0.0	2.3±0.0
		**TF**	61.7±0.8	69.6±2.1	140.0±6.3	16.9±0.4	<LOQ	5.5±0.0	230.9±3.8	513.4±3.7	51.9±1.6	138.4±1.0	1.8±0.1	3.4±0.0	2.7±0.0	0.1±0.0	0.3±0.0	0.8±0.0	6.7±0.2
	**Subplot B, 2019**																		
	**C**	**TV**	59.7±0.7	67.7±0.3	246.6±3.1	82.8±0.8	<LOQ	20.8±0.3	766.8±7.3	619.9±3.6	132.1±2.5	126.7±1.0	1.7±0.1	3.0±0.0	2.8±0.0	0.1±0.0	0.2±0.0	0.2±0.0	2.2±0.0
		**TF**	81.3±1.5	88.4±1.6	114.7±1.1	<LOQ	<LOQ	<LOQ	132.6±1.3	418.4±0.1	32.1±0.2	117.2±2.3	1.2±0.0	2.8±0.0	2.1±0.0	0.1±0.0	0.2±0.0	0.1±0.0	2.6±0.0
	**BABA**	**TV**	50.9±0.9	54.7±1.5	191.3±3.4	25.8±0.9	<LOQ	19.8±0.4	375.0±5.8	566.3±7.6	116.7±0.9	147.8±0.4	1.4±0.1	3.0±0.0	3.5±0.0	0.1±0.0	0.2±0.0	0.2±0.0	3.6±0.0
		**TF**	60.2±0.9	75.7±0.6	89.5±3.4	<LOQ	<LOQ	3.3±0.0	132.1±0.4	480.4±0.9	49.3±1.1	148.5±1.5	1.3±0.1	2.4±0.0	2.1±0.0	<LOQ	0.2±0.0	0.1±0.0	9.3±0.0
	**GT**	**TV**	39.3±0.6	48.5±0.3	132.0±4.3	8.3±0.0	<LOQ	<LOQ	175.6±0.6	379.7±2.1	50.6±1.1	104.9±1.3	1.0±0.0	2.7±0.0	1.8±0.0	0.1±0.0	0.1±0.0	0.1±0.0	1.5±0.0
		**TF**	61.8±0.2	68.0±1.9	70.2±1.1	<LOQ	<LOQ	<LOQ	162.1±2.0	413.2±7.7	27.6±0.7	124.3±0.7	1.5±0.1	3.0±0.0	2.6±0.0	0.1±0.0	0.3±0.0	0.2±0.0	4.5±0.0
	**BF**	**TV**	54.4±0.8	65.7±0.9	134.0±2.3	55.8±0.3	<LOQ	29.6±0.1	442.8±5.0	604.6±3.9	126.0±4.2	174.1±6.1	1.5±0.1	3.0±0.0	3.3±0.0	0.1±0.0	0.3±0.0	0.2±0.0	3.7±0.2

		**TF**	56.9±0.2	63.5±1.6	<LOQ	<LOQ	<LOQ	<LOQ	144.4±3.8	269.5±5.3	27.1±0.6	128.8±3.2	1.6±0.0	2.8±0.0	1.9±0.0	<LOQ	0.2±0.0	0.1±0.0	5.1±0.0
	**ST**	**TV**	50.9±0.3	55.5±1.3	177.3±4.7	17.1±0.1	<LOQ	2.0±0.0	233.7±9.4	410.0±9.2	56.1±0.8	118.5±0.0	1.0±0.0	2.9±0.0	2.9±0.0	0.1±0.0	0.2±0.0	0.2±0.0	3.4±0.0
		**TF**	53.7±1.1	59.2±0.1	103.1±2.1	6.5±0.0	<LOQ	<LOQ	236.1±4.9	426.4±18.3	41.6±1.5	140.3±0.5	1.2±0.1	2.6±0.0	1.6±0.0	<LOQ	0.2±0.0	0.5±0.0	3.9±0.0
	**Subplot A, 2018**																		
**Musts**	**C**	**TV**	15.9±2.5	189.0±0.6	13.0±0.3	22.4±0.2	7.8±0.1	381.0±3.3	588.1±14.3	54.1±0.8	227.9±7.7	0.9±0.0	0.4±0.0	1.9±0.0	1.9±0.0	0.1±0.0	0.1±0.0	<LOQ	1.4±0.0
		**TF**	13.1±0.2	141.6±0.1	19.7±0.3	56.5±0.0	<LOQ	255.3±1.4	554.9±1.7	53.8±0.2	213.7±2.2	1.1±0.0	0.5±0.0	2.9±0.0	0.6±0.0	0.2±0.0	0.2±0.0	<LOQ	1.6±0.0
	**BABA**	**TV**	111.0±0.7	135.0±3.0	17.1±0.1	21.7±0.3	11.2±0.0	220.4±3.6	455.6±10.4	46.6±0.7	169.5±4.0	1.2±0.0	0.2±0.0	2.2±0.0	1.3±0.0	0.1±0.0	0.2±0.0	<LOQ	0.7±0.0
		**TF**	129.4±0.9	136.2±2.5	11.5±0.1	23.1±0.3	<LOQ	118.4±1.2	302.0±2.0	35.0±0.7	132.7±1.9	1.1±0.0	0.2±0.0	2.3±0.0	1.3±0.0	0.1±0.0	0.2±0.0	<LOQ	1.7±0.0
	**GT**	**TV**	131.9±1.7	143.1±3.8	25.7±0.1	35.0±0.1	<LOQ	447.4±0.8	643.8±10.4	57.9±2.6	219.4±0.2	1.4±0.0	0.7±0.0	2.0±0.0	0.7±0.0	0.1±0.0	0.2±0.0	0.1±0.0	6.4±0.0
		**TF**	104.2±2.8	112.4±0.4	21.1±0.7	61.6±1.2	<LOQ	556.9±14.7	1144.2±34.7	123.9±3.9	236.3±6.8	2.9±0.0	0.7±0.0	2.2±0.0	0.7±0.0	0.2±0.0	0.3±0.0	<LOQ	2.4±0.0
	**BF**	**TV**	97.4±1.1	109.7±1.0	12.8±0.1	26.3±0.0	11.8±0.0	495.1±8.5	624.2±15.6	59.4±2.3	206.0±0.5	1.7±0.0	0.8±0.0	1.9±0.0	1.0±0.0	0.1±0.0	0.3±0.0	<LOQ	1.2±0.0
		**TF**	132.0±0.5	151.1±4.2	16.7±0.2	51.8±0.6	<LOQ	212.9±2.7	449.1±4.5	56.9±0.5	311.2±6.1	1.6±0.0	0.4±0.0	2.1±0.0	0.8±0.0	0.2±0.0	0.3±0.0	<LOQ	1.4±0.0
	**ST**	**TV**	118.0±0.7	140.1±1.2	17.1±0.0	22.7±0.3	6.7±0.1	250.1±3.2	366.9±3.2	41.7±0.6	150.7±2.7	0.8±0.0	0.3±0.0	2.4±0.0	1.3±0.0	0.2±0.0	0.2±0.0	<LOQ	0.9±0.0
		**TF**	72.7±1.3	82.9±1.1	7.9±0.0	17.2±0.1	<LOQ	112.7±0.7	396.8±1.6	36.8±0.8	148.4±1.1	1.5±0.0	0.4±0.0	2.1±0.0	0.9±0.0	0.2±0.0	0.3±0.0	<LOQ	2.5±0.0
	**Subplot B, 2018**																		
	**C**	**TV**	<LOQ	<LOQ	12.5±0.2	20.1±0.4	<LOQ	329.5±4.6	728.5±6.0	33.2±0.8	238.5±2.2	0.9±0.0	0.4±0.0	2.3±0.0	1.8±0.0	0.1±0.0	0.2±0.0	<LOQ	6.5±0.0
		**TF**	109.3±2.6	122.4±2.9	23.1±0.4	3.04±0.0	<LOQ	125.9±0.0	387.2±1.5	38.5±0.6	150.5±2.7	1.0±0.0	0.6±0.0	2.3±0.0	1.6±0.0	0.1±0.0	0.2±0.0	<LOQ	1.9±0.0
	**BABA**	**TV**	136.3±0.5	148.7±0.5	20.6±0.4	35.5±0.5	<LOQ	230.3±4.3	606.6±7.8	42.5±0.5	275.6±7.9	0.9±0.0	0.4±0.0	2.1±0.0	1.7±0.0	0.2±0.0	0.2±0.0	<LOQ	1.7±0.0
		**TF**	130.5±4.1	142.9±4.1	14.4±0.1	55.3±0.5	<LOQ	262.9±0.6	572.6±3.1	53.0±0.3	318.4±1.4	3.6±0.0	0.4±0.0	2.0±0.0	1.6±0.0	0.2±0.0	0.3±0.0	<LOQ	3.3±0.0
	**GT**	**TV**	134.2±2.8	137.3±1.7	30.0±0.2	41.0±0.1	<LOQ	352.0±8.5	632.7±14.1	46.9±0.2	261.4±1.6	1.0±0.0	0.3±0.0	2.1±0.0	2.4±0.0	0.1±0.0	0.2±0.0	<LOQ	8.1±0.1
		**TF**	107.0±2.5	136.2±1.4	13.4±0.2	41.7±1.1	<LOQ	168.2±1.2	450.8±2.7	46.2±1.4	229.6±3.0	0.7±0.0	0.5±0.0	2.3±0.1	0.8±0.0	0.1±0.0	0.2±0.0	<LOQ	4.5±0.0
	**BF**	**TV**	135.1±1.4	155.1±1.4	18.0±0.5	27.2±0.4	<LOQ	274.9±1.3	597.7±0.2	49.1±0.4	252.7±1.2	0.9±0.0	0.2±0.0	2.2±0.0	1.1±0.0	0.1±0.0	0.2±0.0	<LOQ	9.7±0.1

		**TF**	115.9±0.8	167.5±3.6	18.0±0.2	47.3±0.6	<LOQ	205.0±3.1	522.3±5.5	47.4±0.6	269.1±1.9	0.8±0.0	0.2±0.0	2.2±0.0	0.8±0.0	0.1±0.0	0.2±0.0	<LOQ	5.6±0.0
	**ST**	**TV**	122.1±3.7	126.5±2.4	19.9±0.2	29.4±0.6	<LOQ	377.6±9.7	665.2±12.1	47.8±1.1	225.6±2.0	2.2±0.0	0.3±0.0	1.3±0.0	0.8±0.0	0.1±0.0	0.2±0.0	<LOQ	1.4±0.0
		**TF**	108.2±3.3	135.1±2.1	10.4±0.1	20.9±0.0	<LOQ	76.5±0.7	319.6±0.4	37.9±1.1	146.7±1.2	1.3±0.0	0.5±0.0	2.1±0.0	0.5±0.0	0.1±0.0	0.2±0.0	<LOQ	2.3±0.0
	**Subplot A, 2019**																		
	**C**	**TV**	108.6±0.4	133.2±4.3	2.1±0.0	2.7±0.0	0.5±0.0	51.2±1.7	158.3±2.4	15.7±0.4	82.9±1.3	<LOQ	0.7±0.0	1.6±0.0	2.1±0.0	<LOQ	0.3±0.0	<LOQ	2.3±0.0
		**TF**	110.0±1.1	121.0±0.4	16.0±0.0	31.4±0.1	5.5±0.0	568.9±1.6	703.7±0.6	84.0±1.5	173.3±2.8	0.7±0.0	1.3±0.0	1.8±0.0	1.5±0.0	0.1±0.0	0.5±0.0	0.0±0.0	2.8±0.0
	**BABA**	**TV**	110.0±0.6	137.7±3.4	6.9±0.1	7.0±0.0	0.7±0.0	82.2±1.0	224.1±0.3	37.8±0.7	116.8±0.4	<LOQ	0.6±0.0	1.2±0.0	1.9±0.0	0.1±0.0	0.3±0.0	0.0±0.0	2.1±0.0
		**TF**	112.4±1.5	128.1±2.6	12.3±0.1	22.8±0.2	1.3±0.0	137.4±0.3	394.8±3.0	56.9±0.4	120.4±0.5	0.3±0.0	0.3±0.0	1.7±0.0	0.9±0.0	0.1±0.0	0.4±0.0	0.0±0.0	2.5±0.0
	**GT**	**TV**	102.2±2.1	135.5±2.1	34.3±1.9	33.2±1.0	2.7±0.0	210.7±2.9	454.3±3.3	87.2±2.1	168.4±0.4	0.7±0.0	0.7±0.0	1.4±0.0	1.4±0.0	0.1±0.0	0.4±0.0	0.0±0.0	2.5±0.0
		**TF**	108.7±1.8	1285.2±28.0	14.7±0.1	20.8±0.4	3.5±0.0	189.6±2.4	526.4±3.2	68.3±2.2	152.7±5.6	0.8±0.0	1.0±0.0	1.6±0.0	1.4±0.0	0.1±0.0	0.3±0.0	0.0±0.0	6.7±0.0
	**BF**	**TV**	102.7±0.7	122.1±1.1	22.0±0.2	23.2±0.2	1.4±0.0	137.7±0.7	339.6±0.2	71.1±1.0	130.7±0.7	0.1±0.0	0.7±0.0	1.7±0.0	2.1±0.0	0.1±0.0	0.5±0.0	0.0±0.0	2.7±0.0
		**TF**	26.1±0.7	39.0±0.7	12.7±0.1	12.4±0.3	2.9±0.0	120.9±2.0	470.8±1.8	52.0±0.8	176.5±2.3	<LOQ	1.1±0.0	1.4±0.0	1.3±0.0	<LOQ	0.3±0.0	0.0±0.0	4.5±0.0
	**ST**	**TV**	107.8±0.9	130.3±4.7	3.6±0.0	7.5±0.2	0.5±0.0	100.2±0.8	249.0±0.6	24.5±0.2	96.7±0.0	0.1±0.0	0.8±0.0	1.9±0.0	1.8±0.0	0.1±0.0	0.5±0.0	0.0±0.0	1.7±0.0
		**TF**	121.1±1.8	141.3±3.5	16.7±0.0	26.2±0.0	3.0±0.0	251.0±1.1	556.0±1.7	74.1±1.1	205.8±3.2	0.2±0.0	0.8±0.0	1.2±0.0	0.9±0.0	0.1±0.0	0.3±0.0	0.0±0.0	3.4±0.0
	**Subplot B, 2019**																		
	**C**	**TV**	125.5±0.6	156.7±5.6	31.6±1.3	70.7±0.4	6.8±0.0	675.4±4.1	707.5±3.6	116.4±5.0	154.6±3.7	0.9±0.0	1.5±0.0	1.9±0.0	1.9±0.0	0.1±0.0	0.6±0.0	0.0±0.0	1.8±0.0
		**TF**	115.2±0.6	141.3±0.5	18.7±0.0	19.5±0.1	2.3±0.0	169.2±0.2	409.8±0.4	52.6±1.1	139.5±1.0	<LOQ	0.6±0.0	1.2±0.0	0.7±0.0	<LOQ	0.2±0.0	0.0±0.0	1.5±0.0
	**BABA**	**TV**	104.8±1.6	113.5±1.5	28.3±0.0	28.8±0.3	6.6±0.1	271.7±3.6	579.2±8.1	86.5±0.7	148.9±2.3	0.2±0.0	0.7±0.0	1.6±0.0	1.1±0.0	0.1±0.0	0.5±0.0	0.0±0.0	12.7±0.1
		**TF**	108.8±2.2	118.8±2.8	12.6±0.1	26.9±0.5	0.3±0.0	170.1±0.4	545.3±0.9	70.4±0.2	168.5±0.2	<LOQ	0.5±0.0	1.6±0.0	0.7±0.0	<LOQ	0.5±0.0	0.0±0.0	16.0±0.0
	**GT**	**TV**	116.2±0.9	139.8±3.7	17.4±0.3	27.6±0.3	2.1±0.0	234.3±2.9	382.7±6.1	60.4±0.1	146.1±2.3	0.1±0	0.7±0.0	1.5±0.0	1.2±0.0	0.1±0.0	0.2±0.0	0.0±0.0	1.7±0.0
		**TF**	100.0±0.0	127.4±4.9	11.4±0.1	19.1±0.0	2.1±0.0	133.6±3.0	374.9±0.4	50.2±1.1	156.6±0.5	<LOQ	0.9±0.1	1.5±0.0	1.2±0.0	0.1±0.0	0.4±0.0	0.0±0.0	2.2±0.0
	**BF**	**TV**	116.6±3.1	127.2±1.7	30.9±0.3	57.7±0.2	14.0±0.1	473.0±1.3	885.7±1.3	178.7±0.3	205.9±4.6	1.2±0.0	0.9±0.0	1.8±0.0	2.2±0.0	<LOQ	0.6±0.0	0.1±0.0	22.5±0.0

		**TF**	109.4±1.7	125.7±3.1	18.7±0.1	23.9±0.1	3.5±0.0	153.6±0.1	522.7±1.2	61.7±1.5	173.8±0.6	<LOQ	0.7±0.0	1.4±0.0	1.2±0.0	<LOQ	0.6±0.0	0.0±0.0	34.0±0.0
	**ST**	**TV**	101.9±1.2	124.6±1.7	13.4±0.2	26.6±0.7	2.0±0.0	277.7±0.7	471.7±4.6	71.2±1.0	160.4±2.2	0.6±0.0	0.6±0.0	1.5±0.0	1.4±0.0	0.1±0.0	0.3±0.0	0.0±0.0	2.2±0.0
		**TF**	104.9±1.9	119.6±0.5	16.1±0.0	27.6±0.3	2.7±0.0	223.7±0.6	529.5±0.8	62.3±1.3	168.2±1.1	0.3±0.0	0.7±0.0	2.1±0.0	0.7±0.0	<LOQ	0.4±0.0	0.0±0.0	2.7±0.0
